# Clinical complications after a traumatic brain injury and its relation with brain biomarkers

**DOI:** 10.1038/s41598-023-47267-6

**Published:** 2023-11-16

**Authors:** Oriol Yuguero, Maria Bernal, Joan Farré, Montserrat Martinez-Alonso, Ana Vena, Francisco Purroy

**Affiliations:** 1 ERLab, Emergency Medicine Research Group, Institute for Biomedical Research Dr. Pifarré Foundation, IRBLLEIDA, Avda. Rovira Roure 80, 25198 Lleida, Spain; 2 Faculty of Medicine, University of Lleida, Avda. Rovira Roure 80, 25198 Lleida, Spain; 3 Clinical Laboratory, University Hospital Arnau de Vilanova, Avda. Rovira Roure 80, 25198 Lleida, Spain; 4 Systems Biology and Statistical Methods for Biomedical Research Group, Institute for Biomedical Research Dr. Pifarré Foundation, IRBLLEIDA, Avda. Rovira Roure 80, 25198 Lleida, Spain; 5 Clinical neurosciences group, Institute for Biomedical Research Dr. Pifarré Foundation, IRBLLEIDA, Avda. Rovira Roure 80, 25198 Lleida, Spain

**Keywords:** Biomarkers, Neurology, Geriatrics

## Abstract

We aimed to find out which are the most frequent complications for patients who suffer a traumatic brain injury (TBI) and its relation with brain biomarker levels. We conducted a hospital cohort study with patients who attended the Hospital Emergency Department between 1 June 2018 and 31 December 2020. Different variables were collected such as biomarkers levels after 6 h and 12 h of TBI (S100, NSE, UCHL1 and GFAP), clinical and sociodemographic variables, complementary tests, and complications 48 h and 7 days after TBI. Qualitative variables were analysed with Pearson’s chi-square test, and quantitative variables with the Mann–Whitney *U* test. A multivariate logistic regression model for the existence of complications one week after discharge was performed to assess the discriminatory capacity of the clinical variables. A total of 51 controls and 540 patients were included in this study. In the TBI group, the mean age was 83 years, and 53.9% of the patients were male. Complications at seven days were associated with the severity of TBI (p < 0.05) and the number of platelets (p = 0.016). All biomarkers except GFAP showed significant differences in their distribution of values according to gender, with significantly higher values of the three biomarkers for women with respect to men. Patients with complications presented significantly higher S100 values (p < 0.05). The patient’s baseline status, the severity of the TBI and the S100 levels can be very important elements in determining whether a patient may develop complications in the few hours after TBI.

## Introduction

Traumatic brain injury (TBI) is a frequent reason for consultation in Emergency Departments and is becoming a growing public health concern and a major cause of mortality and disability worldwide^1^. TBI is a global health problem, with an estimated incidence of 64 to 74 million cases per year worldwide^2^.

In recent years, there has been an increase in the number of patients over 65 years of age suffering from TBI, mostly linked to falls, and a decrease in road traffic accidents leading to a decrease in TBI among younger people^3^. These data vary in developing countries where TBIs are still caused by road traffic accidents^4^.

The definition of TBI states that there is an alteration in brain function or other evidence of brain damage caused by an external force^5^. This definition recognises that symptoms of brain damage may be delayed or even absent, and therefore other evidence of brain damage that can be obtained analytically or by CT scan is required. Sometimes signs of brain damage can be delayed hours after TBI, and some complications can appear up to a few days or even weeks later. In fact, one of the main concerns is the associated cardiovascular, respiratory or endocrine complications that may occur in a patient who has no pathological findings after TBI and within days may suffer a complication^6^. The development of chronic comorbidities after TBI can complicate recovery from TBI, and increase health costs and mortality^7^.

Recovery after TBI in adult patients will be related to the patient’s baseline status before the event or the use of certain drugs, e.g. anticoagulants and antiplatelet agents^8^.

A recent study in patients aged 75–84 years showed high re-consultation rates and low rehabilitation rates after TBI compared to younger patients^9^. Given the changing sociological profile of TBI patients, we believe it is important to conduct further research in this patient group^10^. In this way we will be able to find out what complications they may suffer in the short term. We will thus be able to improve care in Emergency Departments and adapt visit times to requirements, and propose better options for follow-up after discharge. In fact, a European multicentre study^11^ recommends investigating the complications, beyond the physical sequelae, that geriatric patients may develop, given that in a few years they will be the main population affected by TBIs.

Brain biomarkers of TBI are increasingly studied and some of them are already incorporated in some clinical practice guidelines^12^ to support clinical assessment for the diagnosis and prognosis of patients attending emergency departments for TBI.

Biomarkers of brain damage are usually proteins that are either released by neuronal cells or undergo an increase in their concentrations indicating a pathological change. Of these brain biomarkers, S100 and NSE are among the most studied, although in recent years glial fibrillary acidic protein (GFAP) and ubiquitin L1 carboxyl-terminal hydrolase (UCH-L1), have gained importance.

Hence we proposed this study with the aim of finding out which are the most frequent complications for patients who suffer a TBI immediately after and in the days following the TBI and its relation with brain biomarker levels.

## Methodology

### Material and methods

A hospital cohort study with patients who attended the Hospital Emergency Department between 1 June 2019 and 31 December 2020 due to a traumatic brain injury. The Arnau de Vilanova University Hospital is the main Hospital of a health region with 400,000 people as it is the only public hospital in the region. Patients requiring further interventions may be referred to a third-level hospital in other cities, like Barcelona.

### Sample size

According to several studies, 15% of the population included in the study was expected to experience long-term complications^13–15^. A sample size of 504 patients achieved 80% power to detect a difference between the group proportions of 0.1500. The proportion in group 1 (the TBI group) was assumed to be 0.1500 under the null hypothesis and 0.3000 under the alternative hypothesis^16^.

The proportion in group 2 (the control group) was 0.1500. This group was created with patients who had not suffered TBI to find out the baseline levels of biomarkers in a healthy population.

The test statistic used was the two-sided *Z*-test with pooled variance as the standard method in sample size calculation when comparing two independent proportions. The significance level of the test was targeted at 0.0500. The tool used for sample size calculations was PASS software, version 13.

### Inclusion and exclusion criteria

All patients attending the Emergency Department with a grade 1 or 2 (moderate severe) TBI who agreed to participate were included.

Patients with TBI grade 0 (mild TBI) and those who did not give their consent for inclusion were excluded.

Patients with no TBI were included in the control group. Although this is an observational study, a group of patients without TBI was created to observe the biomarker levels in a baseline population.

### Variables

#### Biomarkers

##### S100

S100 is one of the most studied biomarkers in the context of TBI and it has been observed that increases in its serum concentration are related to pathological situations in the central nervous system (CNS) or to alterations in the blood–brain barrier (BBB)^17,18^.

Kinetic models have shown that S100β levels change rapidly in the first days after injury (especially during the 24–48 h after injury) and that therefore, the interpretation of this biomarker is highly influenced by the time elapsed since trauma^19^.

#### Neurospecific enolase (NSE)

 Enolase (2-phospho-D-glycerate hydrolase, EC 4.2.1.11) is a cytoplasmic enzyme involved in glucose metabolism in both neuronal cells and erythrocytes.

NSE has been extensively studied as a biomarker in the acute phase of TBI and many studies show that increased serum levels correlate with mortality and poor prognosis after TBI^20–22^. NSE has a prolonged half-life of around 24 h, which makes it difficult to use for monitoring the time course of injury^23^.

##### Glial fibrillary acidic protein (GFAP)

Glial fibrillary acidic protein is a monomeric filament protein of the cytoskeleton that is expressed in astroglia cells^24^. This protein has also been extensively studied as a biomarker in TBI due to its diagnostic value along with S100β^25^. After TBI, GFAP rises within one hour and reaches maximum concentrations at around 24 h, decreasing progressively during the first week after 48 h of evolution^26^.

Currently, GFAP is not incorporated into any clinical guidelines^27^.

##### Ubiquitin carboxyl-terminal hydrolase-L1 (UCH-L1)

The least studied biomarker is a deubiquitinase found in the cytoplasm of neurons and, although it was initially thought to be exclusive to the CNS, recent studies have shown that it is not^28,29^. UCH-L1 can be detected at the time of injury in the serum of patients, reaching its peak concentration at 8 h, then starting to decline rapidly in levels within 48 h^26^.

#### Clinical variables

Charlson abbreviated comorbidity index, Glasgow coma score (GCS) on arrival and its fluctuation during the stay in the ED, systolic blood pressure, diastolic blood pressure, temperature, heart rate and pupillary disturbance. The use of antiplatelet and/or anticoagulant treatment was also recorded, as well as antihypertensive, antidiabetic or lipid-lowering treatment.

#### Complementary tests

Haemoglobin, platelet and INR levels on arrival, as well as CT scans and their findings.

#### Socio-demographic variables

Age, gender, place of residence and location of TBI, as well as whether polytrauma had been associated with TBI.

#### Management variables

Time of arrival, time of discharge, observation time, and destination upon discharge.

#### Complications 48 h and 7 days after TBI

We reviewed four groups of complications: traumatic, delirium, heart and respiratory. Trauma complications included post-trauma amnesia as well as cephalea and dizziness. Complications were verified by telephoning the patient and/or relative and on the Hospital primary care computer records. Delirium was evaluated using the 4AT scale^30^ for the early detection of delirium. Heart-related complications referred to the onset of heart failure, angina or acute myocardial infarction. Respiratory complications referred to acute respiratory failure and pulmonary thromboembolism.

### Procedures and handling

Extractions were collected from each subject at 6 and 12 h. At six hours post-trauma, five tubes were extracted: a 5 mL EDTA tube for complete blood count, a 4 mL sodium citrate tube for coagulation (INR), and three 7 mL gel-separation tubes for serum, to determine the biomarkers S100, NSE, GFAP and UCH-L1. At 12 h a further two gel-separation tubes for serum were collected for the determination of biomarkers.

The serum and citrate plasma tubes were entrifugeed at 3500 rpm for 12 min at room temperature. The serum was aliquoted and stored at – 80 °C in the biobank (Biomedical Research Institute, Dr. Pifarré Foundation, IRB Lleida) until the determination of biomarkers.

### Biomarkers measurement

We assayed four biomarkers: S100, neuron-specific enolase (NSE), GFAP and UCH-L1. S100 and NSE were measured at the clinical laboratory (Arnau de Vilanova University Hospital, Lleida, Spain) using a quantitative automated immunoassay. S100 and NSE were assayed using a quantitative method based on a sandwich-type electrochemiluminescence immunoassay (ECLIA, Roche Diagnostics, Mannheim, Germany). Samples were incubated in a first reaction with a biotinylated monoclonal S100-specific (or NSE-specific) antibody and a monoclonal S100-specific (or NSE-specific) antibody labelled with a ruthenium complex reaction to form a sandwich complex. Next, a second incubation was performed by incorporating the streptavidin-coated particles, and the complex formed was fixed to a solid phase through the interaction of biotin and streptavidin. These microparticles were then retained by magnetism on the surface of an electrode, which, upon application of an electric current, produced a chemiluminescent reaction. This resulting light emission was measured by a photomultiplier. Sample results were expressed in µg/L. Limit of detection (lowest detectable analyte concentration) was 0.005 µg/L for S100 and 0.05 µg/L for NSE.

Serum GFAP and UCHL-1 were measured in the same laboratory using a sandwich enzyme-linked immunosorbent assay. UCH-L1 and GFAP were assayed using a sandwich ELISA Kit (DuoSet ELISA, R&D Systems). The following protocol was implemented for the test: 96 well microplates were incubated manually with the mouse capture antibody overnight at room temperature against UCH-L1 or GFPA. The following steps were carried out on Triturus automated equipment (Grifols®), incubating the well microplates with a blocking agent for a minimum of one hour. Subsequently, the sample is added and incubated for a period of two hours. Biotinylated detection antibody (two hours’ incubation) and streptavidin conjugated with horseradish-peroxidase (20 min) are then added. Finally, the substrate (hydrogen peroxide and tetramethylbenzidine) is added for 20 min. After this time, the reaction is stopped with sulfuric acid and the optical density is determined at 450 nm using a microplate reader. Each step of the protocol is interspersed with washing steps. Each assay has its own calibration curve.

### Statistical analysis

For quantitative variables, the median and the 25th and 75th percentiles were obtained. Qualitative variables were analysed with Pearson’s chi-square test, and quantitative variables with the Mann–Whitney *U* test. All tests had a significance level of 5%.

A comparative analysis of the patient cohort with respect to the control sample was performed, as well as of the variables in relation to TBI severity.

A multivariate logistic regression model for the existence of complications one week after discharge was performed to assess the discriminatory capacity of the clinical variables. The final model with the variables that showed a significant statistical contribution according to the likelihood ratio test was built using the Boruta algorithm. Figures were constructed to illustrate its calibration and discriminatory capacity. Finally, Kaplan–Meier curves and survival analysis were performed by fitting a multivariate Cox regression model for patient survival during the follow-up of the study in relation to the clinical variables.

### Ethical aspects

The study was approved by the Clinical Research Ethics Committee of the Arnau de Vilanova University Hospital in Lleida (CEIC-1952).

Informed consent was obtained from all subjects or their legal guardian(s) prior to participate in the study. All methods were performed in accordance with the relevant guidelines and regulations.

The processing, communication and transfer of the personal data of all participating subjects complied with the provisions of Spanish Organic Law 3/2018 on Personal Data Protection and Guarantee of Digital Rights (LOPD-GDD 3/2018) and Regulation 2016/679 (EU) of the European Parliament and of the Council of 27 April 2016.

## Results

**Figure 1:**
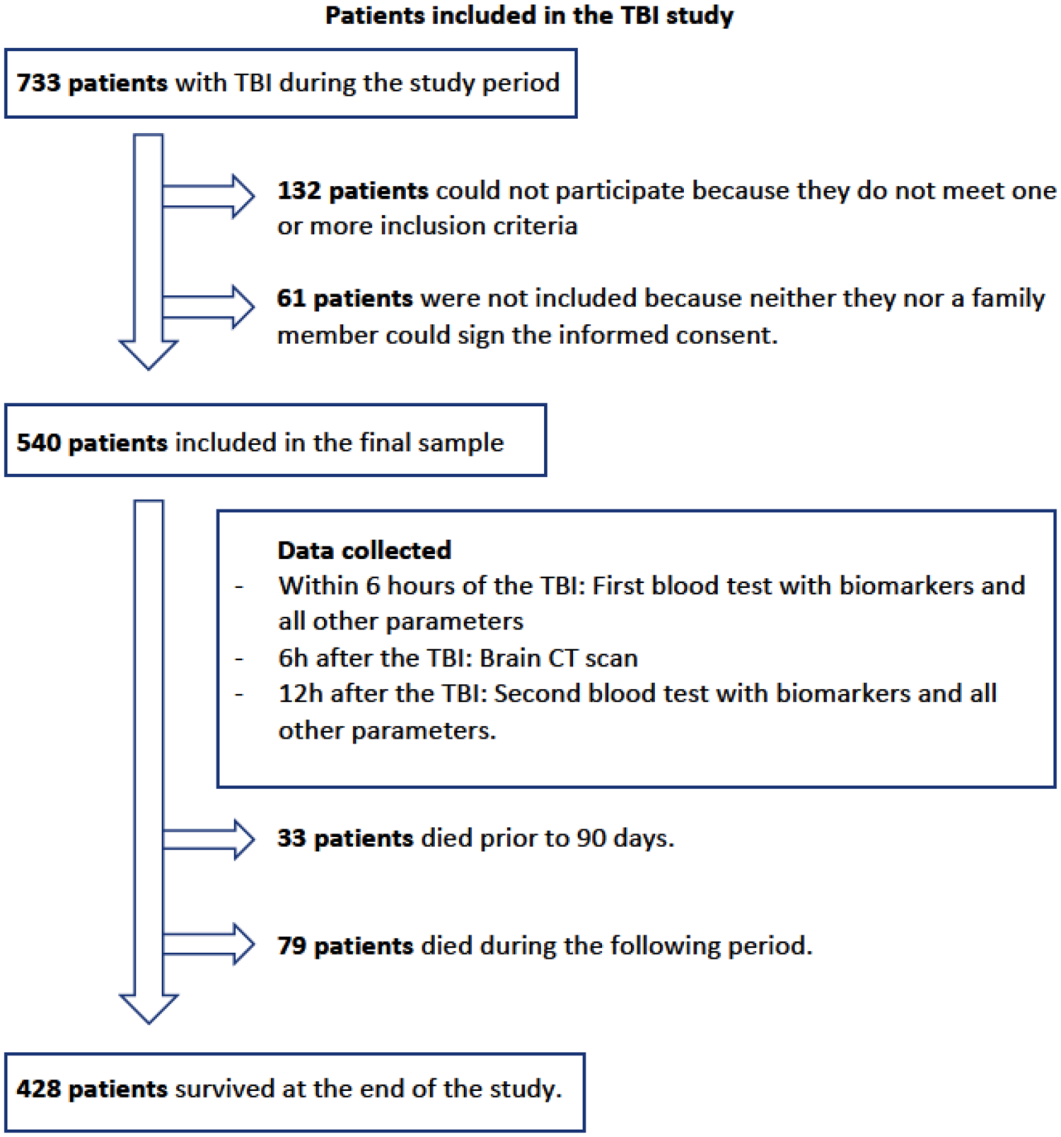
Flowchart of patient inclusion.

A total of 540 patients with TBI aged 17 to 101 were included in this study^16^, together with a subsample of 51 patients without TBI or control group aged 21 to 90 to determine basals levels of biomarkers. In Fig. 1, we can see the flowchart of patient inclusion.

### Biomarkers in TBI vs. non-TBI patients

The comparison of biomarker distribution levels and sex and age distribution between both samples is shown in Table 1.

**Table 1:** Descriptive results for age, sex and biomarkers in controls and TBI patients.

	Controls (n = 51)	TBI patients after 6 h (n = 540*)
Age	32.0 [22.5, 43.0]	83.0 [73.0, 88.0]
Males	9 (17.6%)	291 (53.9%)
S100	0.05 [0.03, 0.06]	0.13 [0.08, 0.25]
NSE	10.4 [8.45, 12.5]	14.8 [11.1, 22.9]
UCHL	3.90 [0.00, 58.0]	0.00 [0.00, 68.4]
UCHL > 0	27 (52.9%)	256 (47.4%)
GFAP	0.00 [0.00, 0.60]	0.00 [0.00, 0.20]
GFAP > 0	25 (49.0%)	149 (27.7%)

*538 non missing values in GFAP.

Given the significant differences between controls and TBI patients in their age and sex distribution, we assessed the sex and age adjusted differences between controls and TBI patients in the median expression of S100 and NSE by using quantile regression. For UCHL and GFAP biomarkers, given their high frequency of undetectable levels (zeros), we assessed the sex and age adjusted odds ratio of detectable levels for TBI patients in comparison with controls by using logistic regression. All regression models assessed the presence of significant interactions between group and the covariates sex and age. There was no significant interaction between group and age Table 2.

**Table 2:** Adjusted differences in biomarkers (TBI vs. control patients).

	TBI vs. controls for women	TBI vs. controls for men
S100 difference	0.10 [0.08, 0.14]	0.07 [0.03, 0.09]
NSE difference	5.58 [3.79, 6.90]	− 0.24 [− 2.30, 2.08]
OR (UCHL > 0)	1.60 [0.70, 3.70]	0.93 [0.21, 3.82]
OR (GFAP > 0)	0.70 [0.29, 1.65]	0.60 [0.14, 2.55]

*Median [95% CI] and OR [95% CI] values adjusted by age and the interaction between sex and group. The quantile regression models for the median difference in S100 and NSE showed, in both cases, a significant interaction between group and sex. The logistic regression models for the log odds of detectable values of UCHL and GFAP did show, in any case, no significant interaction between group and sex but a significant association with age.

The S100 biomarker showed higher age-adjusted median values in TBI patients than in controls, and this difference was significantly higher for women than for men. The NSE biomarker showed higher age-adjusted median values in TBI patients than in controls, but only for women (Table 2). The age-adjusted odds of detectable values for the biomarkers UCHL or GFAP did not significantly differ between TBI patients and controls neither for men nor for women.

### Complications in TBI patients

The Table 3 shows the distribution of the presence of complications at 48 h and 7 days and at any of these times for the sample of patients with traumatic brain injury. The most incident complication during the first 7 days was headache or dizziness, which was present in 20.9% of TBI patients, although only 6.5% still showed it at 7 days assessment. Respiratory and cardiac complications were recorded in 3.7% and 2.8% of patients, respectively, during the first 7 days after the TBI.

**Table 3:** Patients’ complications.

	At 48 h	At 7 days	In 7 days
In-hospital death	5 (0.9%)	7 (1.3%)	7 (1.3%)
Drowsiness	38 (7.0%)	15 (2.8%)	40 (7.4%)
Post-traumatic amnesia	19 (3.5%)	4 (0.7%)	20 (3.7%)
Headache and dizziness	109 (20.2%)	35 (6.5%)	113 (20.9%)
Acute change in the mental state	41 (7.6%)	32 (5.9%)	47 (8.7%)
Easily distracted or difficulty following conversations	29 (5.4%)	19 (3.5%)	34 (6.3%)
Expression of incoherent ideas or conversations	21 (3.9%)	15 (2.8%)	24 (4.4%)
Altered state of consciousness	24 (4.4%)	18 (3.3%)	30 (5.6%)
Respiratory complications	13 (2.4%)	14 (2.6%)	20 (3.7%)
Cardiac complications	9 (1.7%)	8 (1.5%)	15 (2.8%)
Any complication, including in-hospital death	166 (30.7%)	86 (15.9%)	176 (32.6%)

A total of 176 patients (32.6%) showed at least a complication from the ones listed above in the first 7 days after a TBI.

### Identification of predictors of complications in the first 7 days after a TBI

The Table 4 shows the patient’s characteristics in relation with the presence or absence of any complication in the first 7 days after a TBI. In this table we have tried to evaluate the relationship between the occurrence of complications and clinical variables or clinical background.

**Table 4:** Patients’ characteristics in relation with complication in the first 7 days after a TBI.

	All TBI patients	No complication	Complications	OR*	p ratio
Sociodemographic variables					
Age (mean)	83.0 [73.0;88.0]	83.0 [72.0;87.0]	84.0 [73.8;89.0]	1.01 [1.00;1.02]	0.224
Gender N (%)					
Woman	249 (46.1%)	166 (66.7%)	83 (33.3%)		
Man	291 (53.9%)	198 (68.0%)	93 (32.0%)	0.94 [0.65;1.35]	0.735
Place of residence N (%)					
Home	480 (88.9%)	328 (68.3%)	152 (31.7%)		
Geriatric center	56 (10.4%)	34 (60.7%)	22 (39.3%)	1.40 [0.78;2.46]	0.256
Sociosanitary home	4 (0.7%)	2 (50.0%)	2 (50.0%)	2.15 [0.22;20.9]	0.478
Place of TBI: N (%)					
Accident	36 (6.6%)	23 (63.9%)	13 (36.1%)		
Place of residence	387 (71.7%)	256 (66.1%)	131 (33.9%)	0.90 [0.45;1.90]	0.777
Public way	117 (21.7%)	85 (72.6%)	32 (27.4%)	0.67 [0.30;1.51]	0.323
Clinical background					
Charlson comorbidity index (mean)	2.0 [1.0;3.0]	2.0 [1.0;3.0]	2.0 [1.0;3.0]	1.01 [0.90;1.13]	0.874
Dementia: N (%)					
No	440 (81.5%)	295 (67.0%)	145 (33.0%)	0.92 [0.57;1.45]	
Yes	100 (18.5%)	69 (69.0%)	31 (31.0%)		0.714
Hypertension: N (%)					
No	117 (21.7%)	75 (64.1%)	42 (35.9%)	0.83 [0.54;1.28]	
Yes	423 (78.3%)	289 (68.3%)	134 (31.7%)		0.391
Dyslipemia: N (%)					
No	336 (62.2%)	223 (66.4%)	113 (33.6%)	0.88 [0.61;1.28]	
Yes	204 (37.8%)	141 (69.1%)	63 (30.9%)		0.512
Diabetes: N (%)					
No	369 (68.3%)	248 (67.2%)	121 (32.8%)	0.97 [0.66;1.43]	
Yes	171 (31.7%)	116 (67.8%)	55 (32.2%)		0.889
Smoke: N (%)					
No	520 (96.3%)	354 (68.1%)	166 (31.9%)	2.13 [0.85;5.26]	
Yes	20 (3.7%)	10 (50.0%)	10 (50.0%)		0.106
Alcohol intake: N (%)					
No	521 (96.5%)	352 (67.6%)	169 (32.4%)	1.22 [0.44;3.13]	
Yes	19 (3.5%)	12 (63.2%)	7 (36.8%)		0.682
Drug abuse: N (%)					
No	527 (97.6%)	355 (67.4%)	172 (32.6%)	0.94 [0.24;2.98]	
Yes	13 (2.4%)	9 (69.2%)	4 (30.8%)		0.916
Previous treatment N(%)					
Antihypertensive treatment					
No	144 (26.7%)	95 (66.0%)	49 (34.0%)	0.92 [0.61;1.37]	
Yes	396 (73.3%)	269 (67.9%)	127 (32.1%)		0.666
Oral antidiabetic treatment					
No	403 (74.6%)	273 (67.7%)	130 (32.3%)	1.06 [0.70;1.59]	
Yes	137 (25.4%)	91 (66.4%)	46 (33.6%)		0.773
Insulin treatment					
No	495 (91.7%)	335 (67.7%)	160 (32.3%)	1.16 [0.60;2.17]	
Yes	45 (8.3%)	29 (64.4%)	16 (35.6%)		0.653
Statin treatment					
No	377 (69.8%)	248 (65.8%)	129 (34.2%)	0.78 [0.52;1.16]	
Yes	163 (30.2%)	116 (71.2%)	47 (28.8%)		0.222
Anticoagulant treatment					
No	324 (60.0%)	209 (64.5%)	115 (35.5%)	0.72 [0.49;1.04]	
Yes	216 (40.0%)	155 (71.8%)	61 (28.2%)		0.079
Antiplatelet treatment					
No	350 (64.8%)	243 (69.4%)	107 (30.6%)	1.29 [0.89;1.88]	
Yes	190 (35.2%)	121 (63.7%)	69 (36.3%)		0.177
TBI severity, N(%)					
Pupillary alteration					
No	535 (99.1%)	362 (67.7%)	173 (32.3%)	3.06 [0.46;26.5]	
Yes	5 (0.9%)	2 (40.0%)	3 (60.0%)		0.239
TBI code activation					
No	497 (92.0%)	343 (69.0%)	154 (31.0%)	2.33 [1.24;4.40]	
Yes	43 (7.9%)	21 (48.8%)	22 (51.2%)		**0.009**
Coagulation is reversed					
No	525 (97.2%)	357 (68.0%)	168 (32.0%)	2.42 [0.84;7.13]	
Yes	15 (2.7%)	7 (46.7%)	8 (53.3%)		0.100
Severity of TBI					
Mild	505 (93.5%)	350 (69.3%)	155 (30.7%)		
Moderate	20 (3.7%)	10 (50.0%)	10 (50.0%)	2.25 [0.90;5.67]	0.083
Severe	15 (2.7%)	4 (26.7%)	11 (73.3%)	6.04 [2.00;22.8]	**0.001**
CT: Normal	326 (60.4%)	223 (68.4%)	103 (31.6%)		
No normal	214 (39.6%)	141 (65.9%)	73 (34.1%)	1.12 [0.78;1.62]	0.543
CT: No acute pathology	466 (86.3%)	326 (70.0%)	140 (30.0%)		
Acute pathology	74 (13.7%)	38 (51.4%)	36 (48.6%)	2.20 [1.34;3.63]	**0.002**
Clinical variables (mean)					
Systolic blood pressure	144 [129, 163]	144 [129, 166]	142 [126, 162]	1.00 [0.99, 1.00]	0.457
Diastolic blood pressure	77.0 [67.0 86.0]	77.0 [68.0, 87.0]	76.5 [67.0, 85.2]	0.99 [0.98, 1.01]	0.37
Heart rate	74.0 [65.0 86.0]	74.0 [65.8, 86.0]	74.0 [65.0, 87.0]	1.00 [0.99, 1.01]	0.664
Temperature	36.0 [35.5 36.3]	36.0 [35.5, 36.3]	36.0 [35.5, 36.3]	0.95 [0.70, 1.28]	0.74
Hemoglobin	12.9 [11.7, 14.1]	12.9 [11.7, 14.2]	12.9 [11.8, 14.0]	0.98 [0.89, 1.08]	0.644
Platelets	204 [160 246]	207 [170, 257]	191 [150, 232]	1.00 [0.99, 1.00]	**0.015**
INR blood test	1.13 [1.03, 1.67]	1.13 [1.04, 1.78]	1.12 [1.03, 1.51]	0.90 [0.74, 1.10]	0.315

*CT* computed tomography, *INR* international normalized ratio.

*OR odds ratio of any complication in the first 7 days after TBI.

Significant values are in bold.

The odds of any complication are significantly associated to TBI code activation, TBI severity level, the presence of acute pathology according to CT and lower levels of platelets. Previous treatment such as anticoagulant and antiplatelet treatment has no association with complications.

Table 5 shows the relation of biomarkers with complications. We have evaluated biomarkers levels after 6 h of TBI and its change between 6 and 12 h after TBI. The odds of any complication are significantly associated with higher levels of S100 after 6 h of TBI.

**Table 5:** Biomarkers levels in relation with complication in the first 7 days after a TBI.

	All TBI patients	No complication	Complications	OR	p ratio
S100 6 h after TBI	0.13 [0.08, 0.25]	0.12 [0.07, 0.23]	0.16 [0.09, 0.31]	1.42 [1.01, 2.01]	**0.045**
NSE 6 h after TBI	14.8 [11.1, 22.9]	14.8 [11.2, 22.0]	14.8 [11.1, 26.2]	1.00 [1.00, 1.01]	0.162
UCHL. 6 h after TBI	0.00 [0.00, 68.4]	0.00 [0.00, 66.7]	0.00 [0.00, 75.6]	1.00 [1.00, 1.00]	0.209
GFAP. 6 h after TBI	0.00 [0.00, 0.20]	0.00 [0.00, 0.30]	0.00 [0.00, 0.00]	0.99 [0.92, 1.06]	0.804
Change in S100 (6 h–12 h)	0.05 [0.02, 0.14]	0.05 [0.02, 0.13]	0.06 [0.02, 0.17]	0.95 [0.57, 1.56]	0.83
Change in NSE (6 h–12 h)	2.45 [− 0.5, 8.55]	2.50 [− 0.50, 8.20]	2.00 [− 0.7, 9.20]	1.00 [1.00, 1.01]	0.112
Change in UCHL (6 h–12 h)	0.00 [0.00, 12.5]	0.00 [0.00, 9.22]	0.00 [0.00, 19.1]	1.00 [1.00, 1.00]	0.733
Change in GFAP(6 h–12 h)	0.00 [0.00, 0.00]	0.00 [0.00, 0.00]	0.00 [0.00, 0.00]	1.16 [0.82, 1.62]	0.403

Significant values are in bold.

The application of the Boruta algorithm identified as important contributors to discriminate patients with complications from those without, the patient’s characteristics of age, S100 after 6 h from the TBI, TBI severity, NSE after 6 h from the TBI, acute pathology in TC and platelets levels. The same algorithm but applied to the number of complications suffered in the first 7 days after a TBI added to the previous list the important contributors of TBI code activation, anticoagulant treatment, INR blood test, GFAP after 6 h from the TBI, hemoglobin levels, UCHL after 6 h from the TBI and the place of residence.

**Figure 2:**
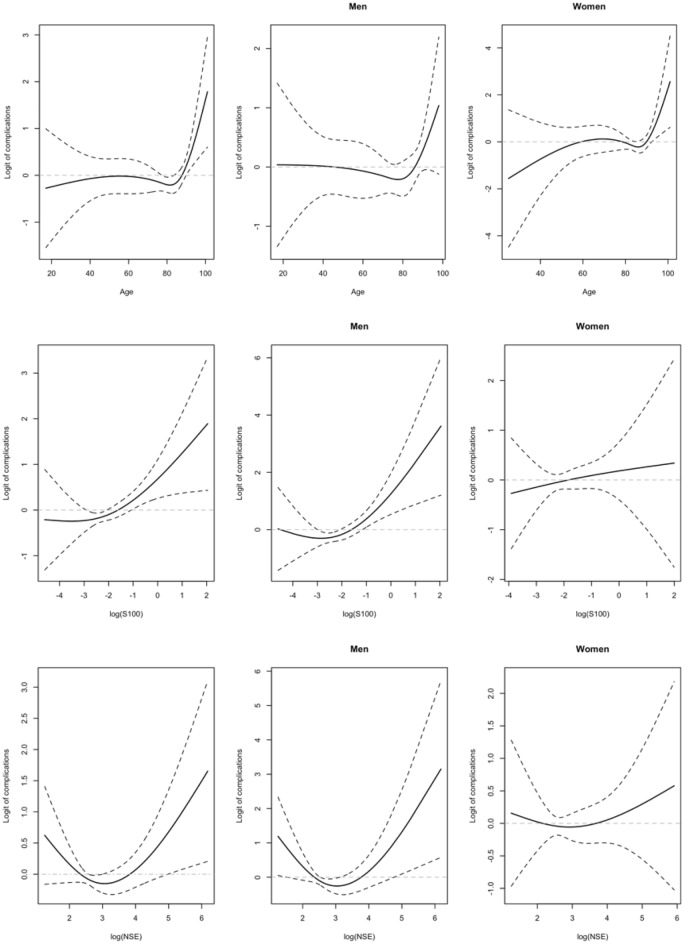
No linearity in the relationship between the logit of complications after the first 7 days from the TBI and the patients’ age and the biomarkers S100 and NSE in natural logarithmic scale, stratified by the sex of the patient. Natural splines with 3 (for age) and 2 (for biomarkers) degrees of freedom were applied.

The assessment of the linearity of the relationship between the quantitative potential predictors and the logit of complications showed a non-linear relationship for age and the biomarkers S100 and NSE after 6 h from the TBI, which also differed by sex for both biomarkers, S100 and NSE levels, as shown in Fig. 2.

According to Fig. 2, there are inflection points common to men and women. The most significant changes are observed for age higher than 84, and S100 and NSE levels higher than 0.08 and 13.2, respectively.

**Figure 3:**
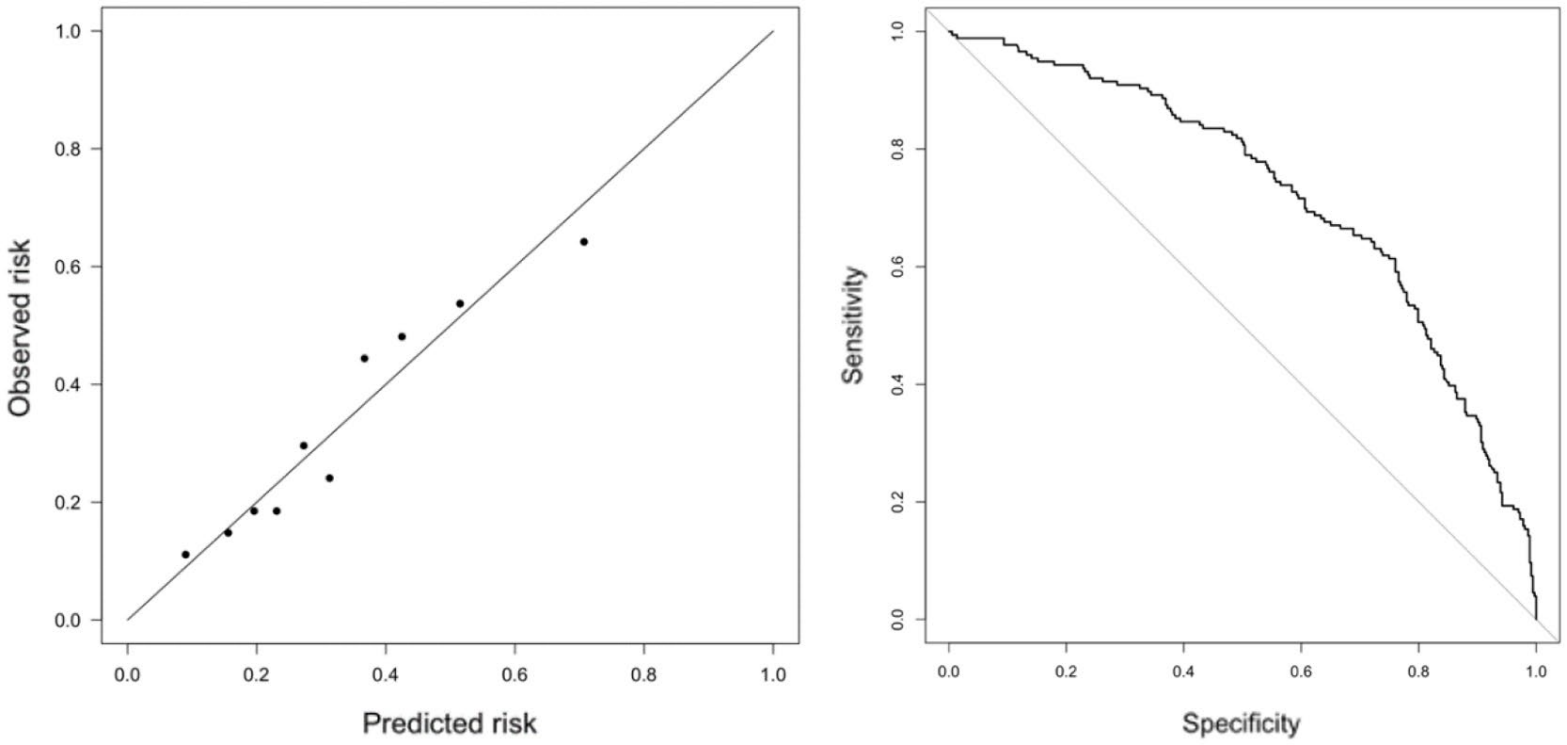
Calibration and discrimination of the multivariable logistic regression model.

The multivariable logistic regression model included the significant predictors of age, TBI severity, platelets levels, TBI code activation, reversed coagulation, treatment with anticoagulants and the biomarkers of S100 and NSE, both in natural logarithmic scale, as well as the significant interaction between sex and S100 levels. The model included also the inflection points identified for age, S100 and NSE. The rest of potential predictors identified by the Boruta algorithm, such as acute pathology in TC, INR blood test, GFAP after 6 h from the TBI, hemoglobin levels, UCHL after 6 h from the TBI and the place of residence did not show a significant contribution to the multivariable model according to the likelihood ratio test and were discarded from the final multivariable model. There was no evidence of a significant lack of calibration (Hosmer–Lemeshow test p-value = 0.68) and the discrimination reached an area under the curve of 0.73, with a 95% confidence interval of [0.68, 0.77], as seen in Fig. 3.

## Discussion

In the cohort of patients suffering TBI, complications were recorded in 30.7% at 48 h after TBI, decreasing to 15.9% of patients after one week. The most frequent complications were headache and/or dizziness after concussion. However, some cardiological or respiratory complications may be triggered by destabilisation after TBI. In our study, we have not detected differences according to the gender of the patients in terms of complications, but we have detected differences in S100 levels and NSE.

Generally we are talking about patients with mild or moderate TBI, but who nevertheless attend the emergency department for evaluation at our hospital shortly after their injury. Consultations by patients with TBI, especially those who are geriatric or who are being treated with anticoagulant drugs, are becoming more frequent.

Patient observation hours in the Emergency Department for this group range between 12 and 24 h, and the resources used vary according to their comorbidities^31^.

Patients who suffer a mild TBI are unlikely to present complications at 48 h and 7 days, despite taking anticoagulant treatment. Moreover, their main complication will be headache. In fact, in one of the models we have developed, patient use of anticoagulants acts as a protective element. Patients receiving anticoagulant treatment are likely to have a better baseline status than others of the same age who do not take anticoagulants. The "protective" role of anticoagulant drugs in the geriatric population as an indicator of good baseline status has already been described by our digestive pathology team^32^. However, if anticoagulation levels need to be reversed (because they are out of range), this suggests an increased risk of complications.

Our results show that the baseline situation of the patients (age, being admitted to a nursing home, especially the more fragile) will condition the complications that may develop after TBI. Fundamentally, patient frailty will be an indicator of possible complications after TBI.

Patients who present moderate severe TBI are more likely to present neurological, cardiac or respiratory complications, and therefore longer observation time or hospital admission is justified in specific cases.

This should lead to the reorganisation of these patients’ circuits^33^, and new tools for home observation using new technologies for patients with mild TBI, thus avoiding transferring them to the hospital, especially in the case of geriatric patients.

In addition, our study shows the relationship between complications and S100 levels, so that on many occasions, the determination of this biomarker could rule out the performance of CT^34^, and thus avoid unnecessary exposure to radiation.

The main strength of our study is its prospective design, with a very representative sample of the TBIs attend to at the emergency departments of medium-sized hospitals. In addition, we have been able to perform a follow-up of several weeks, which allows us to detect the complications that have appeared. We believe that another strength of our study is that we have analysed the 4 main biomarkers that are currently being used in the management of TBI. Few studies combine all 4. In addition, the double measurement at 6 h and 12 h after TBI gives us valuable information on the kinetics of the biomarkers.

However, as limitations we must point out that the complications are self-assessed, since it is the patient or his/her relative/caregiver who identifies the complication, and there may be some observer bias. Moreover, having higher control sample it would be useful to compare the results.

## Conclusions

The use of biomarkers can change the management of some pathologies, such as TBI, from the early stages^35^. Animal models^36^ show a way in which studying the molecular biochemical response after TBI can be interesting in its management^37^. Moreover, there are a huge field of innovation in the management of TBI using proteomics and biomarkers^38^.

In view of the results obtained, our main conclusion is that the patient's baseline status, the severity of TBI and S100 levels can be very important elements in determining whether a patient may develop complications in the few hours after TBI, whether neurological, cardiac or respiratory.

This is the first study to show that the combination of biomarkers and clinical parameters can determine the occurrence of complications in TBI patients. We believe this is really innovative as it could be a breakthrough in reducing radiological tests and waiting times in the emergency department. The use of biomarkers shows a significant change and further studies should be conducted to confirm this change in the management of TBI.

We believe that the most innovative aspect of this article is its clinical and practical implications. If our results are confirmed, we could avoid CT scans in certain patient profiles, and be able to predict the complications that patients may have after a TBI.

## Data Availability

The datasets used and analysed during the current study available from the corresponding author on reasonable request.
